# Metacognitive Monitoring of Text Comprehension: An Investigation on Postdictive Judgments in Typically Developing Children and Children With Reading Comprehension Difficulties

**DOI:** 10.3389/fpsyg.2018.02253

**Published:** 2018-11-20

**Authors:** Chiara Mirandola, Alfonso Ciriello, Martina Gigli, Cesare Cornoldi

**Affiliations:** Department of General Psychology, University of Padua, Padua, Italy

**Keywords:** metacognitive monitoring, text comprehension, postdictive judgments, development, reading comprehension difficulties

## Abstract

The ability to assess and monitor one’s own understanding of a written text is fundamental for learning and academic achievement. In the current paper, postdictive monitoring of text comprehension (i.e., the ability to judge the accuracy of responses previously given to a reading comprehension test) was investigated in both typically developing (TD) children and children with reading comprehension difficulties. Children from primary school (3rd to 5th grade) and secondary school (6th to 8th grade) participated in the study (*N* = 245). They were administered standardized tasks for reading comprehension, in which they had to read two texts and answer 12 multiple-choice questions after each text; subsequently, they had to provide postdictive judgments evaluating their performance: for each answer they had to select whether they judged it as correct, incorrect or whether they were uncertain. Two scores were calculated: Bias score, indicating the difference between metacognitive judgments of accuracy and actual performance; and Accurate estimation, indicating the sum of correct answers judged as “correct” and incorrect answers judged as “incorrect.” Results showed that primary school children were more overconfident than secondary school children and made fewer Accurate estimations especially for “correct” responses. Furthermore, the consideration of a group of children with reading comprehension difficulties showed that these failures are linked to worse metacognitive monitoring ability of comprehension performance in comparison not only to age-matched controls but also to the TD group of third-graders. Implications for learning and achievement are discussed.

## Introduction

Children’s ability to reflect upon the nature of the reading process and to self-assess their comprehension during reading has been largely studied especially in the educational context, over the course of the last 30 years (Garner, 1987; Jacobs and Paris, 1987; Pazzaglia et al., 1995; Roebers et al., 2009). In particular, the ability to monitor one’s own comprehension during text reading is fundamental in everyday life, as well as at school: while reading a text – being it narrative or informative – the child who is able to metacognitively monitor her understanding, becoming aware for example of some unclear passages, will likely be a successful reader and learner.

Up to date, developmental studies have not deeply investigated all the mechanisms involved in the self-assessment of a comprehension test. Nonetheless, this type of monitoring is important during the student’s carrier. As an example, consider a student who just replied to a number of multiple choice questions for her final exam, and has some time to review them before handing in the paper to the teacher. How good is our student at judging the accuracy of her responses? This aspect is particularly important when the child is required to complete frequent comprehension and knowledge tests based on the multiple choice procedure; in many countries, as for example in Italy, this procedure is systematically introduced only with the student’s transition from primary to secondary school, and may contribute to the difficulties that many students meet in this period (Rice et al., 2011). The appropriateness of the postdictive judgements given at the end of a multiple choice test may be crucial in guiding the student to spend the remaining time reconsidering the uncertain responses and eventually changing them. How does this metacognitive monitoring of responses given for complex material such as texts develop with age, especially during the transition from primary to secondary school? Is it linked to reading comprehension abilities? The current paper was aimed at answering these general questions.

Text comprehension is a complex cognitive ability which includes a number of processes and specific skills, such as (1) linguistic competencies, including lexical and syntactic knowledge (Cain and Oakhill, 2006), (2) integration of information derived from the text and prior knowledge (van Dijk and Kintsch, 1983), (3) ability to make inferences (Oakhill et al., 2003), (4) inhibition of irrelevant information (Carretti et al., 2009), (5) readers’ motivation (Guthrie and Wigfield, 1999) and (6) metacognitive abilities (Baker and Brown, 1980). Of importance for the current study, monitoring of text comprehension is a crucial component of metacognition. Metacognition - and specifically procedural metacognition (Nelson and Narens, 1990) – includes two processes, namely monitoring and control. Monitoring is an on-line process that usually enables a person to evaluate elaboration of ongoing information in memory, attention or problem-solving tasks. For example, an individual may evaluate how confident she is to remember a person, an event or an item (*How sure am I that I met this person before?*). In the context of text comprehension, monitoring refers to how effective the students are at judging their own comprehension. If their monitoring is effective and they realize that they did not fully comprehend a passage, then efficient control processes allow them to take action, such as going back to the text and reread such unclear passage. Early research studies on comprehension monitoring employed the error detection paradigm. This procedure investigates the readers’ ability to detect text inconsistencies, including spelling errors, grammatical errors or contradictory sentences (Winograd and Johnston, 1982). It was largely documented that children often fail to detect such text inconsistencies and children with poor reading comprehension abilities are worse than their good comprehenders peers (e.g., August et al., 1984; Otero and Kintsch, 1992; Oakhill et al., 2005).

An alternative way to investigate comprehension monitoring abilities is through postdictive judgments. Postdictive judgements have been largely studied in the context of memory, especially in terms of confidence judgements (e.g., Roebers and Howie, 2003; Ghetti et al., 2008, 2011; Mirandola et al., 2012). For instance, after a study phase, individuals may be administered a recognition memory task and required to identify previously seen target items from distractor items. Afterward, they may be asked to rate how confident they are about their responses at the recognition task. Even if children as young as six can monitor their memory strength through confidence ratings (Ghetti et al., 2002), important developmental improvements occur during middle childhood (Roebers, 2002; Ghetti et al., 2008); furthermore, differences in metamemory monitoring through confidence ratings have been documented in children with developmental disabilities compared to typically developing children (Mirandola et al., 2012). Despite evidence in memory research, postdictive judgments – i.e., the ability to judge the accuracy of responses previously given to a reading comprehension test – have not received much attention in reading comprehension during development (but see Thiede et al., 2009 for a review based on studies with young adults). This is surprising, given that postdictive monitoring of text comprehension is educationally relevant: indeed, children are often administered tests devoted to examine their comprehension (i.e., acquisition) of previously presented texts. If children are aware of “the weakness” of a given response, they can avoid being overconfident and focus on developing strategies to improve accuracy, such as increasing their effort, asking for clarification or -if made possible- rereading the text.

Thus far, research studies have focused on students’ prediction of comprehension accuracy. Individuals may be asked to give Judgments of Learning (JOL) immediately after studying a text or after a delay, estimating how much they think they will be able to answer correctly the later questions. The extant literature shows that children as young as 6 are more efficient at monitoring their memory after a delay than immediately after learning (Schneider et al., 2000), replicating previous findings with adults (Nelson and Dunlosky, 1991). A study investigating monitoring and control processes relative to learning complex material in third and fifth graders showed that both 9- and 11- year-olds have relatively good and similar monitoring ability, but the latter are better at selectively withdrawing answers to a test that later proved to be incorrect, suggesting that control processes are later developing than monitoring processes (Roebers et al., 2009). Furthermore, another study showed that 11- but not 9- year-old children have an efficient metacomprehension accuracy when they have to generate keywords about textual information before providing comprehension ratings (de Bruin et al., 2011), thus even metacognitive monitoring may improve with age, especially when textual material that is educationally relevant is used.

Concerning postdictive judgments, a very recent study showed that although all children were able to differentiate their level of confidence when asked to monitor their performance in a demanding memory task – stating higher confidence for correctly than incorrectly recognized items – 2nd graders did so to a higher extent than kindergarteners (Destan et al., 2017). In another study targeting older children’s monitoring, both 10- and 13- year- old students were found to be poorly calibrated, with a tendency toward overconfidence, when asked to judge their performance in two math problems. However, high achieving students were more calibrated than the low achieving peers, suggesting the importance of the role of metacognitive judgments in academic achievement (Garcìa et al., 2016).

A common aspect of monitoring is *calibration* – or the extent to which individuals are able to provide judgments about their accuracy that correspond to an objective measure of actual performance, such as the comprehension score in a test. An individual is considered well calibrated when her judgments of performance closely match her actual performance. When considering measures of absolute monitoring accuracy, confidence judgments are plotted against the actual proportion of correct answers to the task questions. Positive values indicate overconfidence (i.e., the person overestimated her correct answers, despite actual low performance) whereas negative values indicate underconfidence (i.e., the person underestimated her comprehension performance). An alternative was is to study relative monitoring accuracy and one way to do so is by measuring metacognitive discrimination, i.e., how good is the person at distinguishing between correct and incorrect answers (Roebers and Spiess, 2017). During development, it seems that monitoring incorrect answers is more demanding and more subject to improvements with age than monitoring of correct answers (Roebers et al., 2007; Lyons and Ghetti, 2011). Thus, indexing relative monitoring accuracy by separately analyzing correct and incorrect answers may be informative for the developmental trajectory.

A number of studies found that students tend to be overconfident in their performance, showing to be overoptimistic about their abilities, and this is true in different academic and cognitive domains (Hacker et al., 2008; Lipko et al., 2009; Bol et al., 2010; Garcìa et al., 2016). This finding has important implications for academic achievement and learning outcomes (e.g., Dunlosky and Rawson, 2012), and thus it is vital to understand how it develops throughout the school years, especially during the transition from primary to secondary education. During this period, students have to face important challenges, with respect to developmental, social, cognitive and metacognitive aspects (Eccles et al., 1993; Meneghetti et al., 2007).

Furthermore, special populations of children, including children with reading comprehension disorders, struggle even more than their typically developing peers, especially in the monitoring of their understanding of a written text (Cornoldi, 1990), which in turn affects their successful academic learning. Research has shown a series of weaknesses specific for children with poor text comprehension, including language, working memory, reasoning, vocabulary, etc, but has showed that also metacognition may be implied (Cornoldi and Oakhill, 2013). For example, there is evidence that readers who are poorer and slower at comprehending texts are also less calibrated when post-judging through confidence ratings of their prior performance (Maki et al., 1994). This may be due to a lower accessibility to stored information, which in turn might affect the readers’ confidence in their prior performance (Gernsbacher et al., 1990; Koriat, 1997; Mirandola et al., 2011).

In the current paper we examined the relation between reading comprehension and monitoring asking children of different grades and different comprehension ability to provide postdictive judgments about their text comprehension performance. Children attending primary school (grades 3–5) were compared with students attending the first level of secondary school (grades 6–8 according to the Italian educational system). We decided to focus on this comparison as many school systems assume that the transition from primary to secondary school represents a crucial moment of change in the cognitive and academic growth of children. Including children within this age-range would allow us to detect developmental changes in a period when rapid improvements in metacognition occur. In order to ensure that texts would be of comparable difficulty for children of different grades, we administered standardized reading comprehension texts, specific and appropriate for each grade. Indeed, we wanted to avoid the risk that poor monitoring could be simply due to poor comprehension, a problem that could emerge when using the same material across grades. In this way we could also compare the performance of the participating children to normative data and individuate a group of poor comprehenders. In the first set of analyses we excluded poor comprehenders and compared monitoring abilities of primary vs. secondary school typical readers. We hypothesized that, in general, schooling would produce an increase in monitoring ability but more specifically that poor comprehenders would show a diminished metacognitive monitoring (being less calibrated) than both age-matched and younger typical readers.

## Materials and Methods

### Participants

A group of 267 Italian students attending primary schools (3rd to 5th grade) and secondary schools (6th to 8th grade) participated to this study. Twenty-two students were excluded from the analyses due to diagnosis of neurological disorders or because they did not meet eligible criteria in comprehension performance (please refer to Materials and Procedure section for detail). Thus, the final sample included 245 children. Specifically, primary school children: *n* = 112 (mean age = 115.67 months, *SD* = 10.7; females = 61); secondary school children: *n* = 133 (mean age = 154.1 months, *SD* = 11.4; females = 57). Among these children, 12 primary school children and 15 secondary school children met the criteria for being included in the poor comprehenders group (see section “Result” for detail). Prior to the experiment, written informed consent from the children’s parents was obtained and the study was carried out according the ethical standards and requirements established by the Italian Psychological Association and was approved by the local ethical committee of the University of Padova.

### Materials and Procedure

The study was conducted in one session. Two grade-appropriate texts taken from a standardized battery for reading comprehension tasks (Cornoldi and Carretti, 2016) were collectively administered to the students in their classroom (each class included on average 20 students). The texts range between 226 and 455 words in length, and their length increases with school grade (a different pair of texts is used for each grade). The task’s mean test–retest reliability is 0.58 and its validity has been supported by a series of studies (see Cornoldi and Carretti, 2016). Children were provided with one text and one answering sheet at a time. After reading each text, the students had to answer 12 multiple-choice questions (only one alternative was correct), without any time constraint. Children were allowed to re-read parts of the texts if necessary. At the end of the comprehension task, the texts were collected and children were asked to keep their answer sheet. At this point they were instructed to evaluate their answers. In order to avoid that forgetting would interfere with the metacomprehension task, we administered this last one right after the conclusion of the comprehension test. For each answer, children had to report (a) “+” if they judged their answer as correct; (b) “-” if they judged it as incorrect; (c) “unsure” if they were uncertain about their response accuracy. Participants who did not provide at least 10 comprehension responses for each text (“not answered” was considered also when they ticked two alternatives) and who obtained a comprehension score < 3 for each text were excluded from the analyses. The total duration of task administration was approximately 60 min.

### Scoring

Comprehension performance was evaluated assigning 1 point for each correct answer and 0 for each incorrect answer (the total comprehension score was the sum of the correct answers for each text). For the metacognitive monitoring task, we initially excluded the cases where the child gave a response of ‘uncertainty’ and calculated the following variables : (a) Bias score (indexing calibration) as a measure of absolute monitoring accuracy, which is calculated as the number of items the child thought to have correctly answered minus the actual number of correct responses over the number of test items (24 in our case); (b) Accurate estimation, which is the total number of correct answers that the students accurately judged as correct plus the total number of incorrect answers that the students accurately judged as incorrect. Successively, also the ‘uncertain’ responses were included in the Bias index score considered. This offered not only finer grain data but also a different measure of overconfidence. In fact, we reasoned that the only cases where the child was aware that s/he had a good probability of being wrong was represented by the cases when s/he explicitly made the choice of ‘incorrect’ response: in this case the bias score was obtained by summing to the items associated with a metacognitive estimation of ‘correct’ also the items for which the child gave a response of ‘uncertain’.

## Results

A preliminary analysis on the total sample confirmed that the reading comprehension scores were comparable across grades: indeed, a similar mean number of correct responses ranging from 16 to 18 (out of 24) across the different groups emerged. We then calculated the correlations between comprehension scores and the monitoring measures. The partial Pearson’s correlations, controlling for grade, were significant for all measures: Bias score without uncertain, *r* = −0.53, *p* < 0.001; Bias score with uncertain, *r* = −0.61, *p* < 0.001; Accurate estimation, *r* = 0.27, *p* < 0.001; these correlations indicate that even when controlling for grade, better comprehension abilities were correlated with better calibration (diminished bias score) and higher matching between judged and actual performance.

The subsequent analyses were carried out first on typical readers (average-to-good comprehenders, *N* = 218) and then on readers with comprehension difficulties. We compared the comprehension scores of the current sample with normative data in order to identify poor comprehenders (comprehension score ≤ 15th percentile in the summed score for the two texts). Poor comprehenders (*N* = 27) were compared with a matched group of typical readers.

### Postdictive Judgments in Typical Readers

Table 1 displays the descriptive statistics which refer to the 218 children with reading comprehension scores above the normative 15th percentile. First, we entered the bias score without “uncertain” responses as the dependent measure in an univariate ANOVA with School level as the independent variable. This analysis revealed a significant (although small) main effect of School level, *F*(1,216) = 4.17, *p* = 0.042, $\eta_{p}^{2}$ = 0.02; despite the fact that all children in this sample tended to be underconfident, secondary graders were less overconfident than primary graders (see Table 1).

**Table 1:** Means and standard deviations (in parentheses) of Bias scores, Accurate estimation and metacognitive judgments relative to correct comprehension responses as a function of School level.

	Primary school children	Secondary school children
Bias index without uncertain	−0.09 (0.19)	−0.15 (0.21)
Bias index with uncertain	0.22 (0.14)	0.15 (0.17)
Accurate estimation	0.47 (0.13)	0.53 (0.15)
Correct responses	0.67 (0.11)	0.76 (0.11)
*‘Correct’ judgment* (%)	62 (17)	66 (23)
*‘Incorrect’ judgment* (%)	8 (11)	6 (11)
*‘Uncertain’ judgment* (%)	30 (17)	24 (14)

However, when we calculated the bias score including the “uncertain” responses, and included the score into a similar ANOVA, both groups ended up being overconfident. More specifically, we found a main effect of School level, *F*(1,214) = 11.62, *p* = 0.001, $\eta_{p}^{2}$ = 0.05, such that primary school children were more overconfident than secondary school children. Furthermore, the analysis on Accurate estimation revealed a main effect of School level, *F*(1,216) = 8.98, *p* = 0.003, $\eta_{p}^{2}$ = 0.04, such that secondary school children showed higher Accurate estimation than primary school children (see Table 1).

To further analyze postdictive judgments, we conducted a 2 (School level: primary vs. secondary) × 3 (judgment type: correct vs. incorrect vs. uncertain) repeated measure ANOVA on correct responses. This analysis revealed a main effect of judgment type, *F*(2,428) = 482.57, *p* < 0.001, $\eta_{p}^{2}$ = 0.60, such that overall children gave more ‘correct’ judgements for the answers that indeed turned out to be correct (see Table 1)^1^.

Furthermore, an interaction between School level and Judgment type emerged, *F*(2,428) = 3.13, *p* = 0.045, $\eta_{p}^{2}$ = 0.02; *post hoc* comparisons showed that primary school children produced a higher number of “uncertain” judgments for correct responses than secondary school children (see Table 1). Thus, taken together, our data showed that across school levels, children tended to select the “uncertain” response very frequently, and when the bias score is calculated taking into account these judgments, older children are better calibrated than younger children; in other words, younger children are more overconfident in their comprehension performance than older ones. Furthermore, in secondary school children there is a higher correspondence between postdictive judgments and actual performance (i.e., judging as correct those answers that are indeed correct and as incorrect those answers that are wrong), even if young children demonstrate to have a good metacognitive monitoring too when choosing to a higher extent the “correct” judgment, compared to “incorrect” or “uncertain”, for accurate responses.

### Comparison of Postdictive Judgments in Good vs. Poor Comprehenders

We first compared the monitoring abilities of the group of poor comprehenders (comprehension ≤ 15° percentile) and a group of good comprehenders (comprehension ≥ 70° percentile), matched for numerosity (*n* = 27 within each group), gender (females = 12 within each group), and age (poor comprehenders, *M*age = 137.4 months; *SD* = 19.8; good comprehenders, *M*age = 138.1; *SD* = 20.4). Subsequently we compared the group of poor comprehenders (excluding 4 children attending third-grade) with the group of third-graders (*n* = 37; *M*age = 103 months, *SD* = 3.3) in order to ensure that metacognitive monitoring abilities of poor comprehenders would not be simply comparable to the youngest children in our sample.

Concerning the comparison between poor and good comprehenders, a first univariate ANOVA with Group as the independent variable and bias score (without “uncertain” responses) as the dependent measure revealed a main effect of group, *F*(1,52) = 31.24, *p* < 0.001, $\eta_{p}^{2}$ = 0.375; specifically, good comprehenders underestimated their performance, whereas poor comprehenders tended to overestimate their comprehension. We then considered the ‘uncertain responses’ that resulted more frequent in the case of the poor comprehenders than in the other group; when we included the “uncertain” responses into the bias score, we found a main effect of group, *F*(1,52) = 75.20, *p* < 0.001, $\eta_{p}^{2}$ = 0.60; overall children were overconfident about their performance as they also provided a high number of “uncertain” responses. Nonetheless, poor comprehenders were more overconfident than good comprehenders. Furthermore, the percentage of Accurate estimations was higher for the good comprehenders than poor comprehenders, *F*(1,52) = 4.98, *p* = 0.03, $\eta_{p}^{2}$ = 0.09. See Table 2 for the descriptive statistics. These findings suggest that good comprehenders have a better metacognitive monitoring compared to poor comprehenders, in terms of both better calibration (lower over-confidence) and higher Accurate estimation, i.e., better correspondence between metacognitive judgments and actual comprehension performance.

**Table 2:** Means and standard deviations (in parentheses) of Bias scores and Accurate estimation as a function of comprehension level.

	Good Comprehenders	Poor comprehenders
Bias index without uncertain	−0.21 (0.19)	0.08 (0.24)
Bias index with uncertain	0.10 (0.12)	0.43 (0.10)
Accurate estimation	0.52 (0.15)	0.41 (0.17)

We also wanted to assess whether poor comprehenders’ monitoring abilities would resemble that of younger children. In our case poor comprehenders were distributed across grades as follows: four third-graders, three fourth-graders, five fifth-graders, two sixth graders, ten seventh-graders and three eight-graders (5.74 is the mean grade). In order to have stronger evidence that the monitoring difficulty is specific for the group of poor comprehenders, we compared their performance with that of the youngest group of our typical readers sample, i.e., the group of third-graders. The analysis was carried out excluding the four children with poor comprehension ability attending third-grade and therefore the mean grade of the resulting group is 6.21. The comparisons between poor comprehenders and the group of third graders, revealed a main effect of group for both the bias index without uncertain *F*(1,58) = 7.80, *p* = 0.007, $\eta_{p}^{2}$ = 0.12, (poor comprehenders: *M* = 0.10, *SD* = 0.18; third-graders: *M* = −0.04; *SD* = 0.19) and the bias index including uncertain responses *F*(1,58) = 21.01, *p* < 0.001, $\eta_{p}^{2}$ = 0.26 (poor comprehenders: *M* = 0.41, *SD* = 0.16; third-graders: *M* = 0.23; *SD* = 0.14). These results suggest that the poor comprehenders are more overconfident and thus less calibrated than the youngest group of children. The two groups, however, did not differ in Accurate estimation (poor comprehenders: *M* = 0.44, *SD* = 0.13; third-graders: *M* = 0.46; *SD* = 0.11).

## Discussion

The current study was aimed at investigating metacognitive monitoring of text comprehension through postdictive judgments in both typically developing children and children with reading comprehension difficulties. Evaluating monitoring abilities through postdictive judgments mimics an important testing situation at school and may be crucial during elaboration of the written text. The current findings highlight the important link between comprehension abilities and metacognitive monitoring of texts during development, showing for the first time how young readers with comprehension difficulties underperform in a metacomprehension task using postdictive judgments compared not only to age-matched typical readers, but also to younger ones having the same comprehension abilities. Furthermore, although there is evidence of this link in adults (Thiede et al., 2009), to our knowledge this is the first time that it has been found in young readers during an educationally relevant period of time. All the relevant results will be discussed in turn.

The first important finding of the current study is that – considering the whole sample – comprehension abilities negatively correlated with Bias score and positively correlated with Accurate estimation. Thus, regardless of age, children with better reading comprehension are also more accurate in their metacognitive judgments; indeed they are more calibrated, as shown by the negative correlation with the bias score, and their monitoring closely matches their actual performance. This finding extends to the case of postdictive judgments relative to text comprehension previous observations on poor comprehension and low achievers (Gernsbacher et al., 1990; Maki et al., 1994) and is further qualified by the subsequent analyses on the comparison between good and poor comprehenders.

Typical readers were separated into Primary and Secondary school groups rather than with respect to grade in order to have larger groups, and because we were interested in testing the hypothesis that the transition from primary to secondary school is related with a change in metacognitive monitoring abilities (Eccles et al., 1993; Cornoldi and Oakhill, 2013; Friso et al., 2013). Concerning metacognitive monitoring of typical readers, we must firstly notice that overall children, regardless of school level, tended to select the “uncertain” response very frequently. The initial analysis on the Bias score without uncertain responses, showed that overall children tended to be underconfident, with younger children being less underconfident than older children. However, it is also informative to consider the Bias score including the “uncertain” judgments. Results showed that in this case there was a general tendency to overconfidence but that secondary school students were better calibrated in their monitoring than primary school students, who tended to be more overconfident in their answers; this finding replicates previous evidence - with different paradigms - that age and grade are positively correlated with good monitoring abilities (de Bruin et al., 2011; Destan et al., 2017).

The superior metacognitive monitoring of older children is revealed also in the Accurate estimation measure, showing that secondary school children were better at selecting the “correct” option when indeed their answers turned out to be correct and at selecting the “incorrect” answers when they were wrong. This finding suggests that the transition from primary to secondary school might be crucial for the development of metacognitive monitoring of such a complex activity as comprehending a written text. Previous research investigating metacognitive monitoring and control in a comprehension task and comparing 9- to 11-year-old students (which in the Italian school system correspond to the transition from the last two grades of primary school to the first grade of secondary school) highlighted differences in the control component more than the monitoring one, showing that 11- year- olds are better able than 9- year- olds to withdraw questions that would have been wrong (Roebers et al., 2009). Even if we did not evaluate the control component, we can reason that the development of monitoring processing in a demanding comprehension task – and educationally relevant - might be still poor before the age of 12–13 years. This is also a period when students’ ability to use specific strategies to support reading comprehension is strongly supported by academic achievement, and thus students with higher achievement are also better able to both monitor and control their text comprehension (Meneghetti et al., 2007; Cornoldi and Mirandola, 2014).

Our results on the analyses conducted to evaluate whether children were competent at specifically monitor correct answers and whether this depended on school level shed further light on the development of metacognitive monitoring. Whereas evidence of good monitoring is proven even in younger children in our sample, who indeed selected the “correct” judgment for correct answers more frequently than both “incorrect” and “uncertain” judgments, older children did so to a higher extent, showing that this ability develops with age. This effect was not due to a higher propensity in older children to give a “correct” judgment as students of the secondary school selected to a lower extent, compared to students of the primary school, “correct” judgments for incorrect comprehension responses, a case that appears particularly relevant (Roebers et al., 2007; Roderer and Roebers, 2010; Lyons and Ghetti, 2011) and should be examined in future research introducing comprehension tasks with a higher proportion of difficult questions, likely fostering incorrect responses.

In sum, we found that the transition from primary to secondary schools is associated with a significant improvement in monitoring skills, but if we examine the mean scores of the two school groups and the effect sizes of the differences, we should acknowledge that these differences are relatively modest. However, when we considered monitoring abilities in children with reading comprehension difficulties we found particularly interesting results as the difference between poor comprehenders and grade-matched good comprehenders was very large. It has been argued (Hulme and Snowling, 2009) that when assessing children with learning disabilities, an appropriate comparison should imply children of comparable abilities, in order to examine whether an underlying weakness is simply due to a poorer ability or to specific characteristics of the disabled group. In our case, this analysis was difficult as children were tested with materials appropriate for their grade, and it was not possible to know to which grade corresponded the comprehension abilities of the poor comprehenders’ group. It has been hypothesized that poor comprehenders usually read at a level corresponding to one to two grades below their actual grade. In our case, the mean grade of the poor comprehenders was more than two grades higher than third grade, but – in order to have strong evidence that the monitoring difficulty of poor comprehenders is even below the reading comprehension level – we compared them to the youngest group of our typical readers sample, i.e., the group of third-graders. Results impressively confirmed that a poor comprehension ability is related with a metacognitive monitoring difficulty. Previous evidence on the relation between comprehension and metacognitive monitoring was mixed, with studies showing superior metacomprehension in good compared to poor comprehenders (Maki and Berry, 1984; Maki et al., 1994) and studies showing non-significant correlations (Pressley et al., 1987). However, one important difference among studies is the metacognitive measures used, varying from error detection in texts, predictions of future performance and confidence judgments on past performance.

Thus the present study offers interesting results, but it also has some limitations. For example, the materials could have been the same for all participants, or on the contrary adapted for each child after a preliminary analysis of their comprehension ability. Furthermore, our choice of asking participants to select whether they judged their performance as correct, incorrect or whether they were uncertain poses some limitations: we could not consider these postdictive judgments on a continuum and thus could not calculate gamma correlations as typically done in this research field. However, the correlation between metacognition and performance suggests that the choice of allowing these metacognitive judgments might be a good alternative when testing metacomprehension. Furthermore, the definition of the group of poor comprehenders was simply based only on the performance at the experimental task itself rather than on independent measures (not only of reading comprehension, but also of reading decoding and non-verbal intelligence) as it often happens in the field (Cornoldi and Oakhill, 2013); this assessment was not possible in the present study due to school restrictions. These measures should be collected and investigated in future research.

Despite these limitations, the present findings provide initial evidence that metacognitive monitoring abilities through the provision of postdictive judgments not only improves with age but is also correlated with comprehension abilities; specifically, children with poor reading comprehension abilities are less calibrated when judging their prior understanding of written texts and less accurate in their estimation of performance than both good comprehending peers (matched for age) and younger typical readers. This evidence has important implications: first, metacognitive abilities should receive more attention during daily school activities and in particular during testing situations; second, both typical readers – at a time when metacognitive monitoring abilities rapidly develop – and poor comprehenders would benefit from interventions devoted to the improvement of metacomprehension. For example, there is evidence that both young (Lucangeli et al., 1995; Carretti et al., 2014) and college readers (Thiede et al., 2010) may benefit from an intervention, increasing both text comprehension and metacognition. In particular, in the study of Thiede et al. (2010), students were trained to construct concept maps of a text (i.e., graphical representations of the text’s content which help making connections among different parts of the text) while reading it. The authors argue that the observed improvement in at-risk readers’ performance was due to the increased ability to select better strategies for learning which in turn enhanced the situational model of the text (Kintsch, 1998; Thiede et al., 2010). Metacognitive monitoring should be enhanced through specific training programs and included in educational curricula especially when systematic testing with multiple choice’s options is introduced, as it happens in many countries during the transition from primary to secondary schools. In particular, children should be trained to monitor their responses at the end of the tests and to do it appropriately in order to enhance learning, an aspect neglected in the extant training programs for young readers. This would enhance the probability that students pay more attention to their uncertain responses and spend further cognitive and metacognitive resources to improve them.

## Ethics Statement

This study was carried out in accordance with the recommendations of Italian Psychological Association with written informed consent from all participating children’s parents. All parents gave written informed consent in accordance with the Declaration of Helsinki. The study was approved by the local ethical committee of the University of Padua.

## Author Contributions

CC designed the study. MG and AC collected the data. CM and CC conducted the analyses and wrote the paper with contributions from the other authors. All authors contributed to the data interpretation and approved the final version of the manuscript.

## Conflict of Interest Statement

The authors declare that the research was conducted in the absence of any commercial or financial relationships that could be construed as a potential conflict of interest.

## Footnotes

1 Notice that the effect was not due to an increased tendency of older children to give a ‘correct’ judgment, as in the case of incorrect responses, the tendency to erroneously judge as ‘correct’ the responses was higher for younger children (48% of the cases) than for older children (40%).
